# Progress in Psychiatry

**Published:** 1903-05-09

**Authors:** 


*(To be continued.)*

### Progress in Psychiatry.

**Inherited Syphilis and Insanity.**—Syphilis may act on the impregnated ovum or on the fœtus at different times, with the result that different lesions of the nervous system are produced. Idiocy and imbecility often result from inherited syphilis, and it is now established by a consensus of results obtained by most investigators (Alzheimer, Krafft-Ebing, Thiry) that "juvenile general paralysis," which manifests itself in the period of puberty and adolescence, is the consequence of congenital syphilis. Syphilis attacking the fœtus in the early stages, *i.e.*, as far back as the sixth week of fœtal life, produces diseases or malformations according to the date of infection. As the various organs and tissues of the embryo attain rudimentary development at varying times the effects of syphilis vary correspondingly according to the degree of development of the organ affected by the poison. When syphilis attacks the fœtus it not only affects the bodily organs but also the so-called fœtal appendages—placenta, membranes, and umbilical cord (Ercolani, Fraenkel, Gascard,[1] d'Aulnay,[2] Bissell,[3] Macè, and Durante[4]). The placenta exhibits general pallor and is beset with yellowish-white patches. It is softer than usual, and may even be friable.[5] Microscopically there is diffuse endarteritis and periarteritis with scattered thrombotic areas. The so-called "gummata" of the placenta are probably hæmorrhagic in origin. Patches of endarteritis and thrombi also occur in the vessels of the umbilical cord, and absence of the jelly of Wharton, causing dissociation of the vessels of the cord, has been observed by Macè and Durante. Hydramnios is a common accompaniment, and of the fœtal organs affected (probably *vià* the umbilical cord) the liver first (interstitial hepatitis) and next the lungs show the earliest morbid changes. The various parts of the central nervous system are unequally developed in fœtal life. At a period when the cerebral hemispheres, cerebellum, and ponto-bulb are still rudimentary in structure, the spinal cord has attained a more advanced stage of development. The embryonic encephalon under the influence of the syphilitic virus is liable to undergo nutritive changes which result in dystrophies and anomalies of development, rather than disease in the strict sense of the word. The spinal cord, however, which is more advanced in development exhibits under similar conditions signs of syphilitic meningomyelitis of a diffuse character,[6] a pathological condition resembling in nature the interstitial hepatitis alluded to above. Fœtal syphilis affecting the embryo after it has secured an attachment to the mother by means of its placenta and membranes, ought perhaps to be distinguished from syphilis affecting the impregnated ovum while the latter is still an unsegmented cell or has not as yet developed beyond the stage of a rudimentary blastoderm. For it is probable, judging from analogy, from experimental evidence on teratology (Driesch,[7] Binet[8] and others), and from zoological observations on the embryology of the lower vertebrata, that noxious physico-chemical agencies and toxins (in which category we must include the virus of syphilis), acting on the organism in its embryonic period of life, produce aberrations and monstrosities of development of organs—changes of a very different character from those met with in the middle and later periods of fœtal infection when inflammatory, thrombotic, hæmorrhagic and neoplastic lesions are the result, inasmuch as the organs have already reached some stable degree of morphological differentiation and development. Such infants die within a few days of birth, and usually within a month. Syphilis affecting the fœtus at or about the middle period of fœtal life (the fourth and fifth months) tends to produce disease of the placenta and membranes as well as organic visceral disease of lesions within the fœtus. Such placental and somatic changes are to some extent compatible with intra-uterine life, but they seriously interfere with extra-uterine life, for under the changed circumstances of birth into the world (circulatory, respiratory, digestive, assimilatory and metabolic) the demands made on the bodily organs are much greater, and the visceral lesions render life under the new conditions impossible. Such children when born at full term or prematurely are liable to develop pemphigus of the soles and palms which readily become filled with a blood-stained and purulent exudate, followed by the formation of irregular ulcers. Syphilitic infection of the fœtus in the last two months of pregnancy does not manifest itself by definite signs until the latter half of the second month of infancy. There is no adenitis as in acquired syphilis, and a roseolous rash is rare. When infection occurs at or near full term the syphilitic manifestations which reveal themselves in the latter half of the second month after birth are a roseolous rash, rhinitis of a refractory character, and soreness and redness about the arms and buttocks. As regards the mechanism of syphilitic infection, therefore, the possibilities are (*a*) damage of the ovum or spermatozoon at the period of impregnation; (*b*) syphilitic affection of the embryo prior to the development of a placenta and fœtal circulation, and attended with dystrophies and developmental anomalies; (*c*) syphilitic infection of the fœtus in mid-term with resulting grave visceral lesions which tend to a fatal issue soon after birth; and (*d*) syphilitic infection at or near full term, with consequent manifestations (rash, rhinitis, etc.) towards the end of the second month of extra-uterine life. The central nervous system may thus sustain all degrees of damage varying from arrested and abnormal development (anencephaly, idiocy, imbecility) to diffuse meningo-encephalitis and focal destructive neuro-vascular lesions manifesting themselves in childhood and early life as forms of cerebral paralysis (with or without epilepsy), gross neuropathic defects accompanied by bodily "stigmata" of degeneracy, and juvenile general paralysis of the insane. The dystrophies and bodily stigmata of inherited syphilis may be tabulated as follows according to Fournier[9]:—

Dystrophies and Bodily Stigmata Resulting from Inherited Syphilis.

I. *General Dystrophies.*—(1) Simian and senile ("wizened old man") physiognomy; (2) Persistent infantile condition of the body; (3) Osteogenic exostoses and rickets.

II. *Partial Dystrophies.*—(1) Cranial malformations (microcephaly, hydrocephaly, plagiocephaly, cranio-facial asymmetry, synostoses); (2) Dental-maxillary dystrophies (microdontism, absence of certain teeth, malformation of the jaws); (3) Hare-lip and cleft palate; (4) Ocular and aural dystrophies (strabismus, coloboma, deformed auricles); (5) Spinal scoliosis and spina bifida; (6) Dystrophy of limbs and digits (partial gigantism, micromaly, polydactyly, syndactyly, congenital club-foot); (7) Cerebro-spinal dystrophies, including deaf-mutism; (8) Cardio-vascular defects (congenital cyanosis); (9) Hernia, imperforate anus, and other anomalies of the alimentary tract; (10) Genito-urinary anomalies (ectropion of bladder and testicles, epispadias, cryptorchidy, malformations of the uterus and vulva); (11) Cutaneous dystrophies (ichthyosis, alopecia, nævi, scleroderma, and dermoid cysts); (12) Monstrosities (exomphalos, anencephaly, pseudencephaly, meningocele).

III. *Dystrophies of General Health.*—(1) Hæmophilia, paroxysmal hæmoglobinuria; (2) General or local obesity; (3) Tubercle, Little's disease; (4) Epilepsy, infantile convulsions.

[1] Thèse de Paris, 1885. [2] Archives de Tocologie, 1894. [3] American Jour. of Gynæcol. and Obstetrics, 1897, p. 147. [4] Archives de Gynæcol., 1895. [5] Ballantyne, the Pathology of the Fœtus, 1900. [6] Nouvelle Iconographie de la Salpêtrière, 1896, ix., p. 80. [7] Driesch, Entwickelungsmechanik. [8] Discussion of Alcoholism and Heredity, Brit. Med. Jour. (Proceedings of Section of Psychology), 1898. [9] Fournier, Les Affections Parasyphilitiques.

